# Urinary miR-16-5p can be used as a potential marker of endocapillary hypercellularity in IgA nephropathy

**DOI:** 10.1038/s41598-023-32910-z

**Published:** 2023-04-13

**Authors:** Meng Zhang, Zhi-Yu Duan, Qiu-Yue Zhang, Xie-Guan-Xuan Xu, Yan Zhang, Peng Wang, Shu-Wei Duan, Jie Wu, Xiang-Mei Chen, Guang-Yan Cai

**Affiliations:** 1 Medical School of Chinese PLA, Beijing, 100853 China; 2 Department of Nephrology, First Medical Center of Chinese PLA General Hospital, Nephrology Institute of the Chinese People’s Liberation Army, State Key Laboratory of Kidney Diseases, National Clinical Research Center for Kidney Diseases, Beijing Key Laboratory of Kidney Disease Research, Beijing, 100853 China

**Keywords:** Biomarkers, Nephrology

## Abstract

The most prevalent primary glomerulonephritis and leading cause of end-stage renal disease worldwide is IgA nephropathy (IgAN). More and more studies are describing urinary microRNA (miRNA) as a non-invasive marker for a variety of renal diseases. We screened candidate miRNAs based on data from three published IgAN urinary sediment miRNAs chips. In separate confirmation and validation cohorts, we included 174 IgAN patients, 100 patients with other nephropathies as disease controls (DC), and 97 normal controls (NC) for quantitative real-time PCR. A total of three candidate miRNAs, miR-16-5p, Let-7g-5p, miR-15a-5p were obtained. In both the confirmation and validation cohorts, these miRNAs levels were considerably higher in the IgAN than in NC, with miR-16-5p significantly higher than in DC. The area under the ROC curve for urinary miR-16-5p levels was 0.73. Correlation analysis suggested that miR-16-5p was positively correlated with endocapillary hypercellularity (r = 0.164 p = 0.031). When miR-16-5p was combined with eGFR, proteinuria and C4, the AUC value for predicting endocapillary hypercellularity was 0.726. By following the renal function of patients with IgAN, the levels of miR-16-5p were noticeably higher in the IgAN progressors than in the non- progressors (p = 0.036). Urinary sediment miR-16-5p can be used as noninvasive biomarkers for the assessment of endocapillary hypercellularity and diagnosis of IgA nephropathy. Furthermore, urinary miR-16-5p may be predictors of renal progression.

## Introduction

IgA nephropathy (IgAN), the most prevalent primary glomerulonephritis world-wide, causes end-stage renal disease (ESRD) in 30–40% of patients with biopsy-proven IgAN, making it the major reason of ESRD in many areas around the world. In addition, this disease is particularly common in Asian populations, accounting for 40–50% of primary glomerulonephritis^1^. Renal biopsy is the primary technique for confirming the diagnosis of IgAN, which is an invasive test that is not easily repeated and cannot be performed in many hospitals. Therefore, it is important to find non-invasive biomarkers that can diagnose IgAN.

Endogenous non-coding RNAs known as microRNAs (miRNAs) have a length of 19–25 nucleotides and are significant regulators of gene expression. They achieve this by promoting the breakdown of mRNA and/or blocking its translation. MiRNAs are readily available, relatively stable in form, and not easily degraded^2^. Urine sediment has the advantages of being non-invasive, painless, and can be repeatedly retained, and has a variety of exfoliated cells, including podocytes, renal tubular epithelial cells, immune cells, and stem cells in urine, which can be used to explore cellular changes in the damaged kidney. As a result, discovering non-invasive indicators of IgAN using urine sediment miRNAs may be a novel method^3^.

At this stage, most studies on biomarkers related to miRNAs in IgAN have used miRNA microarray for screening, and the obtained target miRNAs was scattered and lack systematic review. Most studies included only IgAN patients and normal controls. The purpose of our present research was to review the results of previous related studies to obtain target miRNAs. Other nephropathies that are not IgAN were also included. To research the correlation between miRNAs and the pathological and clinical indicators of IgAN, as well as any potential mechanisms involved.

## Methods

### Overall study design

**Figure 1:**
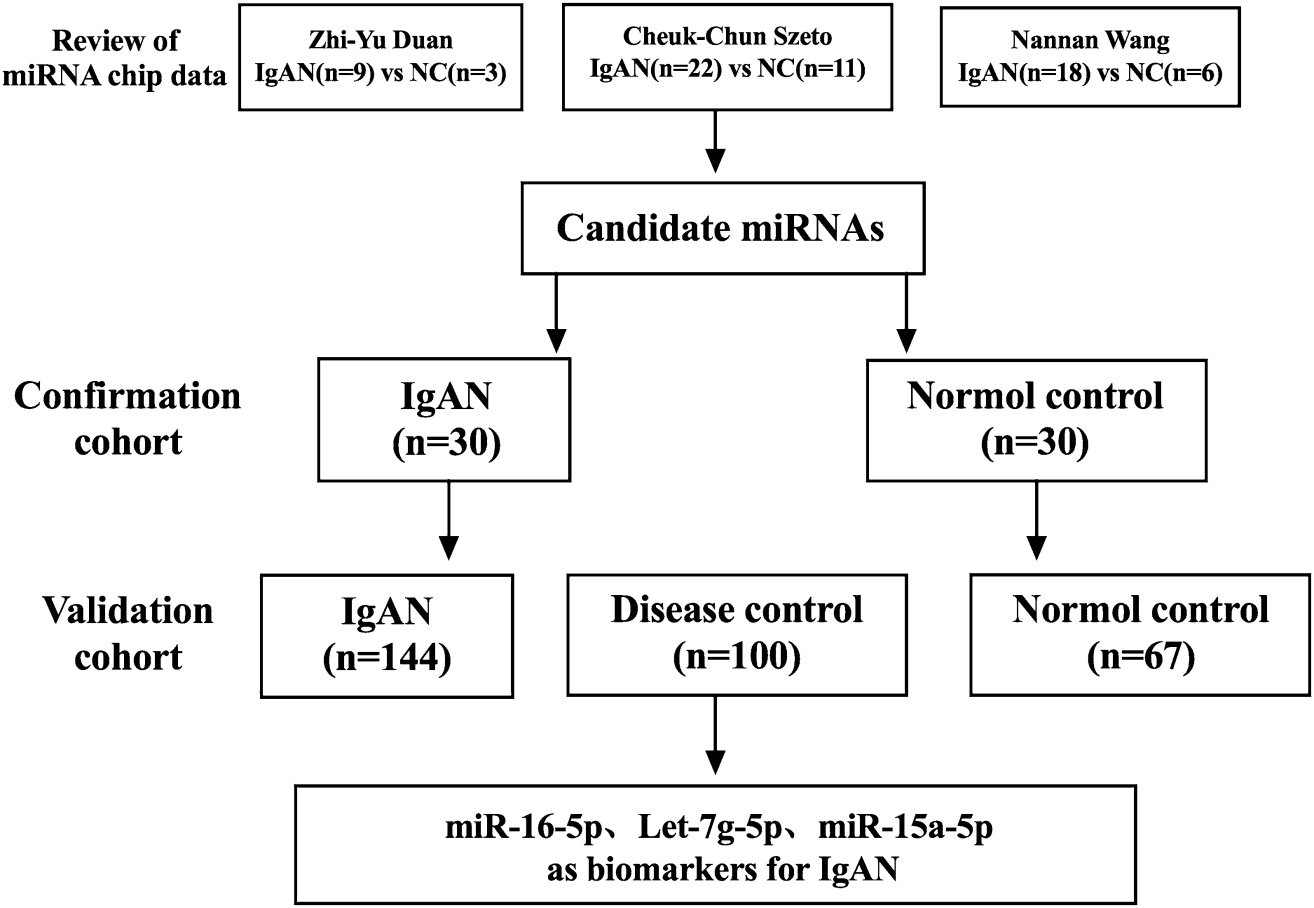
The discovery on IgAN biomarkers.

In this research, we reviewed a total of three profiles of urinary sediment miRNAs in the literature on IgAN by Zhi-Yu Duan, Cheuk-Chun Szeto, and Nannan Wang^4–6^, and found that miR-16-5p, Let-7g-5p, and miR-15a-5p were expressed in the same trend in the three miRNAs profiles, all of them were significantly higher in IgAN compared with normal controls, thus identifying the target miRNAs for this study, as shown in Fig. 1. This study included two separate patient cohorts. First, in the development cohort, we included 30 patients with biopsy-proven IgAN from October 2018 to February 2019 and 30 NC as controls for small sample validation. In the validation cohort, a total of 144 patients with IgAN, 100 DC and 67 NC from March 2019 to May 2022 were included for further extended validation.

### Enrollment of subjects

Patients who had their initial kidney biopsies at the Department of Nephrology, First Medical Center of Chinese PLA General Hospital from October 2018 to May 2022 were consecutively recruited for this study. A total of 174 cases of IgAN, 63 cases of membranous nephropathy (MN), 8 cases of minimal change disease (MCD), 16 cases of focal segmental glomerular sclerosis (FSGS), and 13 cases of diabetic nephropathy (DN) were included according to the diagnosis confirmed by renal biopsy. We excluded 51 patients, 17 patients because of fewer than 8 glomeruli in light microscopic, 2 patients with ESRD prior to renal biopsy, and we also excluded patients with concurrent diagnosis of two or more renal diseases (n = 4), secondary IgA nephropathy (n = 13) (e.g. hepatitis-associated glomerulonephritis, systemic lupus erythematosus, allergic purpura, psoriasis, dry syndrome), and infections of the gastrointestinal, respiratory, or urinary tracts (n = 15). The normal controls were medical workers and routine physical examination patients from the First Medical Center of Chinese PLA General Hospital who had normal kidney function, normal routine urine results, and no personal or family history of kidney disease or other serious illnesses. At the time of the kidney biopsy, all patients had their demographic and clinical information, including their age, sex, mean arterial pressure (MAP), serum creatinine (Scr), and 24-hours urine protein excretion (UPE) recorded. A formula developed by the Chronic Kidney Disease Epidemiology Collaboration (CKD-EPI) was used to calculate glomerular filtration rate (eGFR)^7^.

### Assessing renal histopathology damage

Two qualified pathologists graded the pathological sections of kidney biopsies from IgAN patients using the Oxford classification scoring system^8^. (1) Mesangial hypercellularity (M0/1): 50% of mesangial area with more than three cells is M1; (2) Endocapillary hypercellularity (E0/1): absent or present; (3) Segmental glomerulosclerosis (S0/1): absent or present; (4) Tubular atrophy or interstitial fibrosis (T0/1/2): <25% is T0, >25% to 50% is T1, >50% is T2; (5) Cellular or fibrocellular crescent (C0/1/2): C0 for no crescent, C1 for <25%, and C2 for >25%.

### Sample preparation

Collect 50–100 ml of urine sample from the patient early in the morning on the day of kidney biopsy, place it in a 4 °C refrigerator for temporary storage after collection, and process it within 6 hours. Before use, the supernatant and urine sediment were divided into 50 ml and 1.5 ml EP tubes, respectively, and kept in a −80 °C refrigerator.

### RNA extraction and real-time quantitative PCR

Following the manufacturer's instructions, total RNA from urine sediment was extracted using TRIzol (Invitrogen, USA). Using NanoDrop One^c^ spectrophotometer (Thermo Fisher Scientific, USA), the acquired RNA's concentration (ng/ul) and purity (A260/280) were evaluated. 500ng RNA was used as the system, using miRcute Plus miRNA First-Strand cDNA Kit (Tiangen Biotech, Beijing, China) and Applied Biosystems Veriti^TM^ 96-well Thermal Cycler (Applied Biosystems, USA) for reverse transcription. The housekeeping gene U6 was employed for miRNA detection^9^, and all primers were purchased from Tiangen Biotech. Urine sediment hsa-U6, hsa-miR-16-5p, hsa-Let-7g-5p, and hsa-miR-15a-5p were quantified using miRcute Plus miRNA qPCR Kit (SYBR Green) (Tiangen Biotech, Beijing, China) and Bio-Rad cfx96 real-time system (CFX96, Bio-Rad, USA). The relative quantitative ΔΔCt technique was used to determine the variations in each target miRNA's expression levels in the samples.

### Clinical follow up

After renal biopsy, the patient's renal function is followed up for at least 12 months. According to the KDIGO guidelines, progressor IgAN patients are those with a yearly drop in eGFR more than 5 ml/min/1.73 m^2^, and vice versa for non- progressor^10^.

### Statistical analysis

For graphing and plotting, both SPSS 26.0 and GraphPad Prism 9.4.1 for MAC were used. According to the situation, data were presented as mean ± standard deviation or median (inter-quartile range). The 2^(−ΔΔCt)^ relative quantification method was used to compare the expression of miRNAs. Shapiro-Wilk tests were performed on normality analysis. For normally distributed data, the t test or one-way ANOVA were employed. For data which weren't normally distributed, the Mann-Whitney or Kruskal-Wallis rank test was applied. Correlation analysis was performed using the Pearson or Spearman methods to explore the association between miRNAs and clinical and pathological indicators. The sensitivity and specificity of miRNAs for the diagnosis of IgA nephropathy were calculated utilizing receiver operating characteristic (ROC) curves and area under the ROC curves (AUC). P < 0.05 was used to define statistical significance for the results. All computations were performed with two-tailed probabilities.

### Ethical approval

The studies involving human participants were reviewed and approved by Research Ethics Committee of Chinese PLA General Hospital (approval number S2018-206-01). The patients/participants provided their written informed consent to participate in this study.

## Results

### A review of diagnostic markers of miRNAs in urinary sediment of IgA nephropathy

In this study, we reviewed and compared a total of three profiles of miRNAs in the urinary sediment of IgAN by Zhi-Yu Duan and Nannan Wang from the research team of the Department of Nephrology, First Medical Center of Chinese PLA General Hospital, and Cheuk-Chun Szeto from the research team of the Chinese University of Hong Kong, as shown in Table 1. The expression trends of miR-16-5p, Let-7g-5p, and miR-15a-5p were found to be consistent in the three expression profiles, and all were noticeably higher in IgAN than in normal controls with at least a 10 fold-change, according to Table 2.

**Table 1:** Comparison of the three miRNA profiles from the existing research. IgAN, IgA nephropathy; NC, normal controls; DC, disease controls; MN, membranous nephropathy; MCD, minimal change disease; FSGS, focal segmental glomerular sclerosis; HSPN, Henoch-Schonlein purpura nephritis; RA, renal amyloidosis.

First author	Chip manufacturers	Chip sample size	Number of differential miRNAs	Validation sample size
Cheuk-chun szeto	NanoString technologies	IgAN:22 NC:11	39 IgAN vs NC	IgAN:33 NC:9
Nannan wang	Affymetrix	IgAN:18 NC:6 DC:8(MN4, MCD4)	117 IgAN vsNC 78 IgAN vs MN 11 IgANvs MCD	IgA:102 MN:41 MCD:27 NC:34
Zhi-Yu DUAN	Agilent	IgAN:9 NC:3	214 IgAN vs NC	IgA:93 MN:15 MCD:5 FSGS:8 HSPN:5 RA:2 NC:82

**Table 2:** Fold change analysis of miR-16-5p, Let-7g-5p, miR-15a-5p from three miRNA profiles. Fold change: IgAN group/normal controls.

	Cheuk-Chun Szeto	Nannan Wang	Zhi-Yu DUAN
miR-16-5p	58.234	37.25	265.93
Let-7g-5p	32.064	10.72	102.23
miR-15a-5p	17.288	20.08	298.92

### Clinical and pathological features of patients with IgA nephropathy

We included a total of 174 patients with IgAN, 100 disease control patients and 97 healthy individuals. The clinical and demographic details of the study participants are summarized in Table 3. The pathological features of the IgAN group are listed in Table 4.

**Table 3:** Baseline demographic and clinical characteristics of patient groups. IgAN, IgA nephropathy; DC, Disease control; NC, Normal control; GFR, Estimated glomerular filtration rate.

Group	Confirmation cohort		Validation cohort		
	IgAN	NC	IgAN	DC	NC
no. of subjects	30	30	144	100	67
Sex (M: F)	17:13	17:13	78:66	55:45	33:34
Age (year)	36.30 ± 9.75	36.92 ± 10.27	36.51 ± 10.42	43.72 ± 14.19	38.50 ± 9.16
Proteinuria (g/day)	0.96 (0.81–1.41)	–	1.35 (0.78–2.22)	3.33 (1.71–5.45)	–
Serum creatinine (umol/L)	97.55 (78.60–133.30)	–	99.25 (80.30–125.18)	73.85 (62.03–113.83)	–
eGFR (ml/min/1.73m^2^)	70.17 ± 28.98	–	72.00 ± 27.44	97.55(60.94–114.11)	–

**Table 4:** Pathological characteristics of patients with IgAN.

	Confirmation cohort	Validation cohort
no. of subjects	30	144
M0/M1 (n)	18:12	77:67
E0/E1 (n)	22:8	118:26
S0/S1 (n)	9:21	24:120
T0/T1/T2 (n)	18:12:0	92:46:6
C0/C1/C2 (n)	25:5:0	88:56:0

### Urinary miR-16-5p level in the cohorts

**Figure 2:**
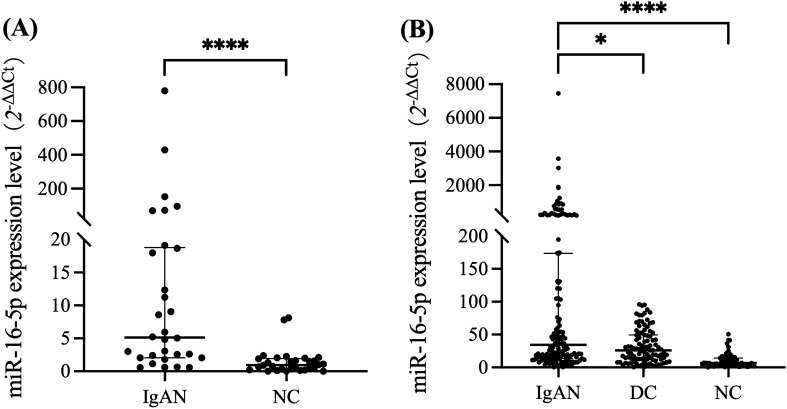
(**A**) Comparison of miR-16-5p expression levels between the IgAN group and normal group in a confirmation cohort. IgAN group, n = 30, Normal control, n = 30. (**B**) Comparison of miR-16-5p expression levels between the IgAN group and Disease control group and normal control group in a validation cohort. IgAN group, n = 144, Disease control, n = 100, Normal control, n = 67. *, P < 0.05; ****, P < 0.0001.

First, in the confirmation cohort, we validated three miRNAs in 30 patients with IgAN and 30 normal patients. Patients with IgAN had significantly greater levels of miR-16-5p expression in urine sediment when compared to the normal controls, P < 0.0001, with a statistically significant difference (Fig. 2A). To further clarify whether these urine sediment markers are specific for the diagnosis of IgAN, we included 100 disease controls including MN, FSGS, and DN in the validation cohort. Urine sediment miR-16-5p differed significantly from both disease controls and normal controls (P = 0.0201,P < 0.0001) , according to Fig. 2B. The expressions of Let-7g-5p and miR-15a-5p in the two cohorts were shown in Supplementary Fig. 1.

### Diagnostic value of miR-16-5p

ROC curves were used to examine the effectiveness of urine miRNA levels in the diagnosis of IgA nephropathy. The area under the curve (AUC) of miR-16-5p, Let-7g-5p, and miR-15a-5p ROC curves were all statistically significant, miR-16-5p had the best diagnostic value (AUC = 0.73, 95% CI 0.679–0.781, P < 0.0001) (Table. 5). When the optimal cut-off value of miR-16-5p was 0.324, the positive predictive value (PPV) was 63.3% and the negative predictive value (NPV) was 68.4%.

**Table 5:** Parameters of the receiver operating characteristic curve and predictive value of miRNAs for diagnosing IgAN. AUC, the area under the ROC curve; CI, confidence interval; PPV, positive predictive value; NPV, negative predictive value. IgAN group, n = 174, Disease control ,n = 100, Normal control, n = 97.

	miR-16-5p	Let-7g-5p	miR-15a-5p
AUC	0.73	0.58	0.70
95% CI	0.679–0.781	0.526–0.643	0.646–0.753
P value	P < 0.0001	P = 0.006	P < 0.0001
Optimal cut-off value	2^−ΔCt^ = 0.324	2^*−*ΔCt^ = 0.0064	2^*−*ΔCt^ = 0.0468
Sensitivity (%)	65.9	57.1	72.9
Specificity (%)	66.1	55.7	58.9
PPV (%)	63.3	52.4	60.8
NPV (%)	68.4	58.7	71.1

### Correlation between miR-16-5p and endocapillary hypercellularity

**Figure 3:**
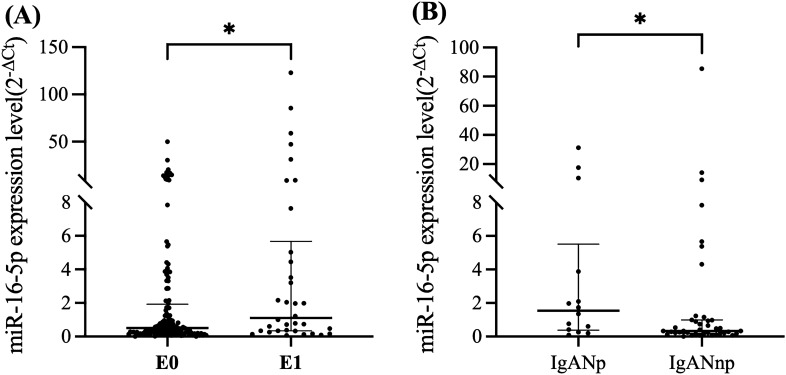
(**A**) Levels of urinary miR-16-5p in patients with different grade of Endocapillary hypercellularity. (**B**) Comparison of urinary sediment miR-16-5p expression levels between the IgAN progressors and non-progressors. IgANp (progressor), IgANnp (non-progressor).

Through correlation analysis, we found that miR-16-5p had no significant correlation with clinical indicators such as eGFR (r = -0.144 p = 0.058) and proteinuria (r = 0.018 p = 0.811), while in terms of pathological indicators, miR-16-5p in urine sediment was positively correlated with endocapillary hypercellularity (r = 0.164 p = 0.031). Patients with E1 classification of IgAN had considerably greater levels of miR-16-5p in urine sediment than E0 classification[1.013(0.335-5.676) vs 0.512(0.224–1.918)] (Fig. 3A). In addition, endocapillary hypercellularity was significantly negatively correlated with blood complement C4 (r = -0.188 p = 0.013), the blood complement C4 of IgAN with E1 was significantly lower than that of E0 [23.85(18.98–27.55) vs 26.35(22.53–30.83)]. Finally, the complete correlation analysis data of the three miRNAs were shown in supplementary materials (Supplementary Table 1).

### Prediction values of miR-16-5p in endocapillary hypercellularity

According to ROC analysis, the AUC values of miR-16-5p, eGFR, proteinuria and C4 for predicting IgAN E1 grade were 0.619, 0.600, 0.480 and 0.363, respectively. miR-16-5p had the highest value for predicting E1 grade alone. When miR-16-5p was combined with eGFR, proteinuria and C4, the AUC value for predicting endocapillary hypercellularity was 0.726, and the predictive value was improved from the previous one.

### Correlation between miR-16-5p and renal progression

To confirm the efficacy of urine sediment miRNAs in predicting the development of renal function in IgAN, 122 IgAN patients were followed for a mean of 8.5 (4–18) months, with a total of 54 IgAN followed for >12 months, 14 of whom were progressors, including one patient who developed ESKD with eGFR ≤15 ml/min/1.73m^2^, and 40 patients in the non- progressors. The level of miR-16-5p was considerably greater in the progressor than in the non-progressor (P = 0.036) (Fig. 3B), suggesting that the higher the baseline miR-16-5p expression, the faster the decline in eGFR.

## Discussion

In this study, we identified three miRNA targets that differed significantly in urine levels between IgA nephropathy and normal controls. Among them, urinary miR-16-5p is a potential marker of endocapillary hypercellularity in IgA nephropathy, which has a good diagnostic value and may be a predictor of renal function progression in IgA nephropathy.

Previous research has looked into the differences in miRNA levels in the urine of people with IgAN^4–6^. For example, Wang et al.^6^ reported that miR-3613-3p expression was significantly downregulated in the urine of IgAN patients and miR-3613-3p and miR-4668-5p correlated with disease severity. MiR-25-3p, miR-144-3p, and miR-486-5p in urinary sediment may be diagnostic markers of IgA nephropathy and are mainly derived from urinary red blood cells. Higher levels of miR-144-3p expression are linked to lower urine protein and better renal function^4^. Similarly, Liang et al.^11^reported urinary miR-21 and miR-205 as prospective prognostic markers for the assessment of interstitial tubular damage in IgAN, and urinary miR-204 was reported as a diagnostic tool for IgA nephropathy^5^. Therefore, we reviewed the urinary sediment miRNA profile published in previous studies on IgAN and found that miR-16-5p, Let-7g-5p, and miR-15a-5p expression trends were consistent in three studies^4–6^, all of which were noticeably increased in patients with IgAN in contrast to normal controls, with a fold change > 10, thus identifying the target miRNAs for this study. The PCR results of the confirmation cohort showed significant differences between IgAN and normal patients, totally supporting the trend of the microarray study discussed above. We then included disease controls for non-IgAN in an independent validation cohort, and the results suggested that urinary miR-16-5p may be a diagnostic biomarker of IgAN. Analysis of the ROC curve revealed good specificity and sensitivity.

In addition to the diagnostic value, we further investigated the clinical significance of these urinary sediment miRNAs. Proteinuria, eGFR and Oxford classification were all closely associated with the progression and prognosis of IgAN^12–14^. In our results, miR-16-5p levels were significantly higher in the urine sediment of patients with E1 in contrast to E0 patients, and blood complement C4 levels were lower in E1 compared with E0 patients, further confirming that endocapillary hypercellularity is a hallmark of active inflammation^15^ and that endothelial cell proliferation is primarily the result of inflammatory cell infiltration^16^. Bao et al.^17^used mesangial cells to stimulate endothelial cells, and their miRNA microarray results showed high expression of miR-16-5p. Therefore, we hypothesized that under galactose-deficient IgA1 stimulation, mesangial cells secreted substances that stimulated endothelial cells to overexpress miR-16-5p. Studies have demonstrated that in patients with IgAN who are not receiving immunosuppressive therapy, endocapillary hypercellularity is a reliable indicator of the rate of renal function loss^18^. Thus the lack of prognostic value for the E score in other studies is likely the result of immunosuppression-related treatment bias^19,20^. A number of subsequent studies have provided evidence for immunosuppressive treatment of intracapillary proliferative IgAN^21,22^. In addition, Let-7g-5p was positively correlated with baseline proteinuria and negatively correlated with eGFR. The higher the expression level of Let-7g-5p, the more its 24-hour urinary protein and the worse its renal function, suggesting that Let-7g-5p may be associated with prognosis. miR-15a-5p was not associated with proteinuria, eGFR, or pathological indicators. The follow-up of renal function in IgAN patients revealed significant differences in miR-16-5p expression levels between the progressor and non- progressor, all of which suggest that urinary sediment miRNAs may be related to the progression and prognosis of IgAN.

Although the biological functions of miR-16-5p, Let-7g-5p, and miR-15a-5p have not been clarified, bioinformatics analysis may elucidate the roles of these miRNAs by predicting hub genes. A total of 133 common target genes of miR-16-5p, Let-7g-5p, and miR-15a-5p were obtained from database miRDB and starBase. By Gene Ontology (GO) enrichment analysis^23^, the Kyoto Encyclopedia of Genes and Genomes (KEGG) pathway analysis^24,25^ and the Protein-Protein Interaction (PPI) network construction^26,27^, the hub gene CCND1 may have a significant impact on how these miRNAs regulate the biological process of IgAN (Supplementary Figs. 2–4, unpublished). According to GO analysis, the functions of these miRNAs are mainly enriched in the regulation of catabolic, metabolic and renal water homeostasis. Antidiuretic hormone (AVP) is important for water homeostasis, and it has been shown that copeptin, a reliable and stable alternative to circulating AVP, was found to correlate with disease severity and prognosis in IgAN patients by detecting copeptin^28^. According to KEGG pathway enrichment analysis, these miRNAs were significantly enriched in FoxO signaling pathway, mTOR signaling pathway, and Wnt signaling pathway. The FoxO signaling pathway has been shown to play an important role in diseases associated with altered B-cell proliferation and/or activation^29^, while up- and down-regulation of some B cells was indeed found by studies of peripheral blood cell subsets in IgAN^30^. Activation of the mTOR signaling pathway causes activation of downstream signaling proteins such as p70s6K, leading to extracellular matrix deposition in glomerular mesangial cells, renal tubular and collecting duct epithelial cells, renal fibroblast activation, and renal interstitial fibrosis. Rapamycin, as an inhibitor of the mTOR signaling pathway, can effectively inhibit glomerular mesangial cell proliferation and extracellular matrix secretion by early low-dose application, reduce IgA deposition, and protect renal function^31^. It has also been demonstrated that excessive Wnt signaling pathway activation contributes to the development of IgAN^32^. In addition, A critical gene that controls the cell cycle is CCND1, and turning on its expression can encourage cell growth. One study found that aberrant miR-320 expression in IgAN urine and kidney tissue promoted B cell proliferation and increased the expression of CCND1^33^. In a rat model of IgAN, autophagy is inhibited, while the mTOR/S6k1 pathway is activated and CCND1 expression is increased^34^, all of which provide directions for further studies.

Our study has some shortcomings. First, all samples were from a single center and were Chinese, whereas the prevalence of IgAN and disease characteristics vary considerably among different countries and ethnic groups, and further validation is still needed. Second, in this study, the basic demographic and clinical information (e.g., age, renal function, or proteinuria) of patients with IgAN was not exactly matched to the diseased controls and normal controls, and a strict pairwise analysis was not performed due to differences in the number of subjects. In addition, the cellular origin of miRNAs in urine sediment is not clear. miR-16-5p, Let-7g-5p, and miR-15a-5p were reported to be the top 20 miRNAs in human blood erythrocytes^35^, so we speculate that these miRNAs may be derived from erythrocytes, but further experiments are still needed to verify them in urine. Finally, how miR-16-5p, Let-7g-5p, and miR-15a-5p act on IgAN via CCND1 remains to be further explored.

In conclusion, in the first part of the study, we reviewed and validated a significant rise in miR-16-5p, Let-7g-5p, and miR-15a-5p in IgAN. Then, we found a significant correlation between miR-16-5p and endocapillary hypercellularity. Finally, we evaluated the effect of miR-16-5p on the progression of IgAN renal function and made some bioinformatic speculations. These findings support the idea that miR-16-5p is a potential marker reflecting endocapillary hypercellularity and predicting IgAN progression.

## Supplementary Information

Supplementary Information.

## Data Availability

The raw date supporting the conclusions of this article will be made available by the authors, without undue reservation. Correspondence and requests for materials should be addressed to G.-Y.C.
