# Supplementary material for: Urinary miR-16-5p can be used as a potential marker of endocapillary hypercellularity in IgA nephropathy
Source: Sci Rep. 2023 Apr 13;13:6048. doi: 10.1038/s41598-023-32910-z (PMC10101996; doi:10.1038/s41598-023-32910-z)
Supplement: Supplementary file 1 — Supplementary Information. [file 41598_2023_32910_MOESM1_ESM.pdf]

Urinary miR-16-5p can be used as a potential marker of endocapillary hypercellularity in IgA nephropathy

Meng Zhang[1, 2], Zhi-Yu Duan[1, 2], Qiu-Yue Zhang[1, 2], Xie-Guan-Xuan Xu[1, 2], Yan Zhang[2], Peng Wang[2], Shu-Wei Duan[2], Jie Wu[2], Xiang-Mei Chen[2] and Guang-Yan Cai[2]*

[1] Medical School of Chinese PLA, Beijing, 100853, China

[2]Department of Nephrology, First Medical Center of Chinese PLA General Hospital, Nephrology Institute of the Chinese People's Liberation Army, State Key Laboratory of Kidney Diseases, National Clinical Research Center for Kidney Diseases, Beijing Key Laboratory of Kidney Disease Research, Beijing, 100853, China

*corresponding author email: caiguangyan@sina.com

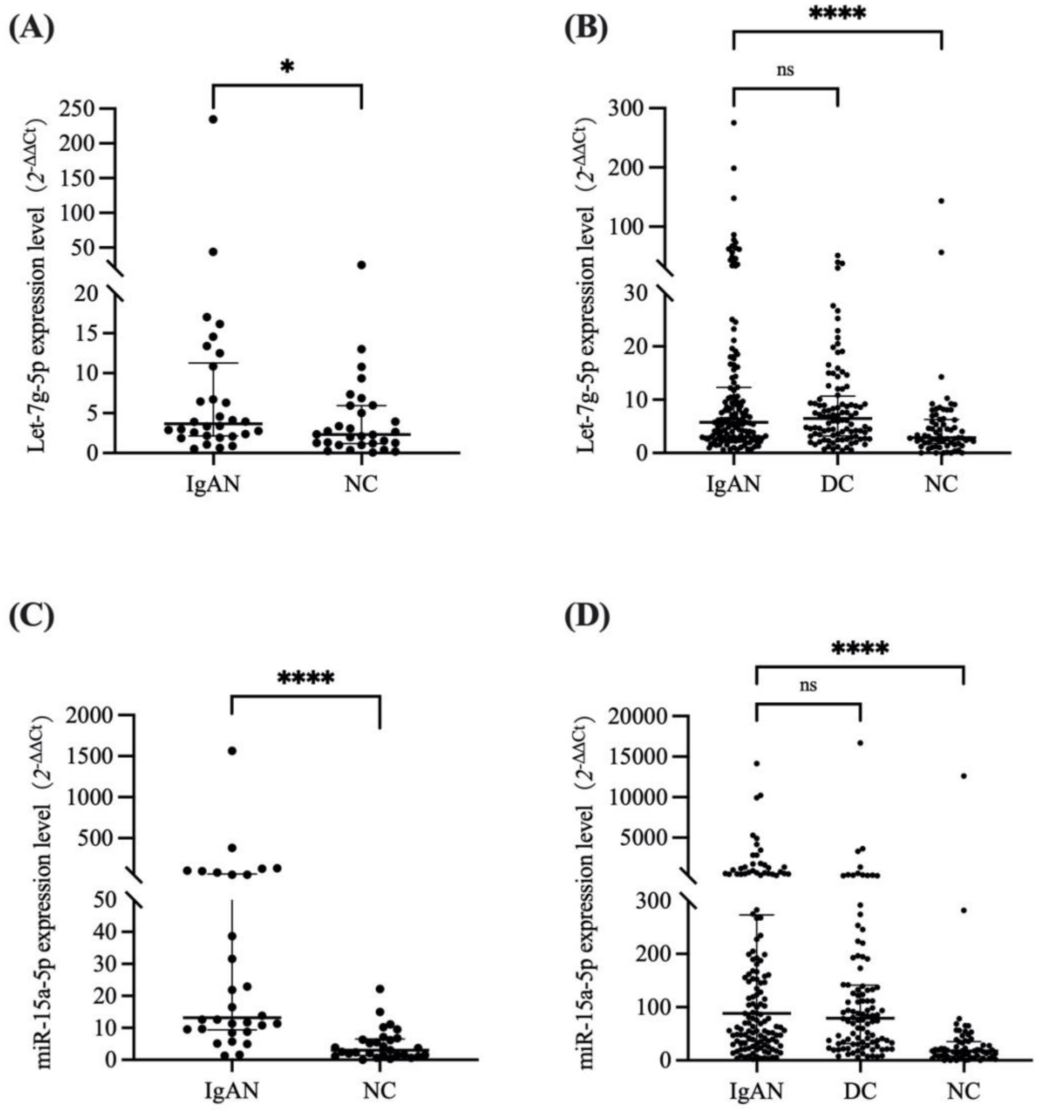

Supplementary Figure 1. Comparison of Let-7g-5p and miR-15a-5p expression levels in cohort. **(A)**Comparison of Let-7g-5p expression levels in confirmation cohort. IgAN, n =30， NC, n =30. **(B)** Comparison of Let-7g-5p expression levels in validation cohort. IgAN, n =144， DC, n=100， NC, n =67.

(C) Comparison of miR-15a-5p expression levels in confirmation cohort. IgAN, n =30， NC, n =30. **(D)** Comparison of miR-15a-5p expression levels in validation cohort. IgAN, n =144，DC, n=100，NC, n =67. IgAN, IgA nephropathy; DC, Disease control; NC, Normal control. *, P< 0.05; ****, P< 0.0001, ns, no significance.

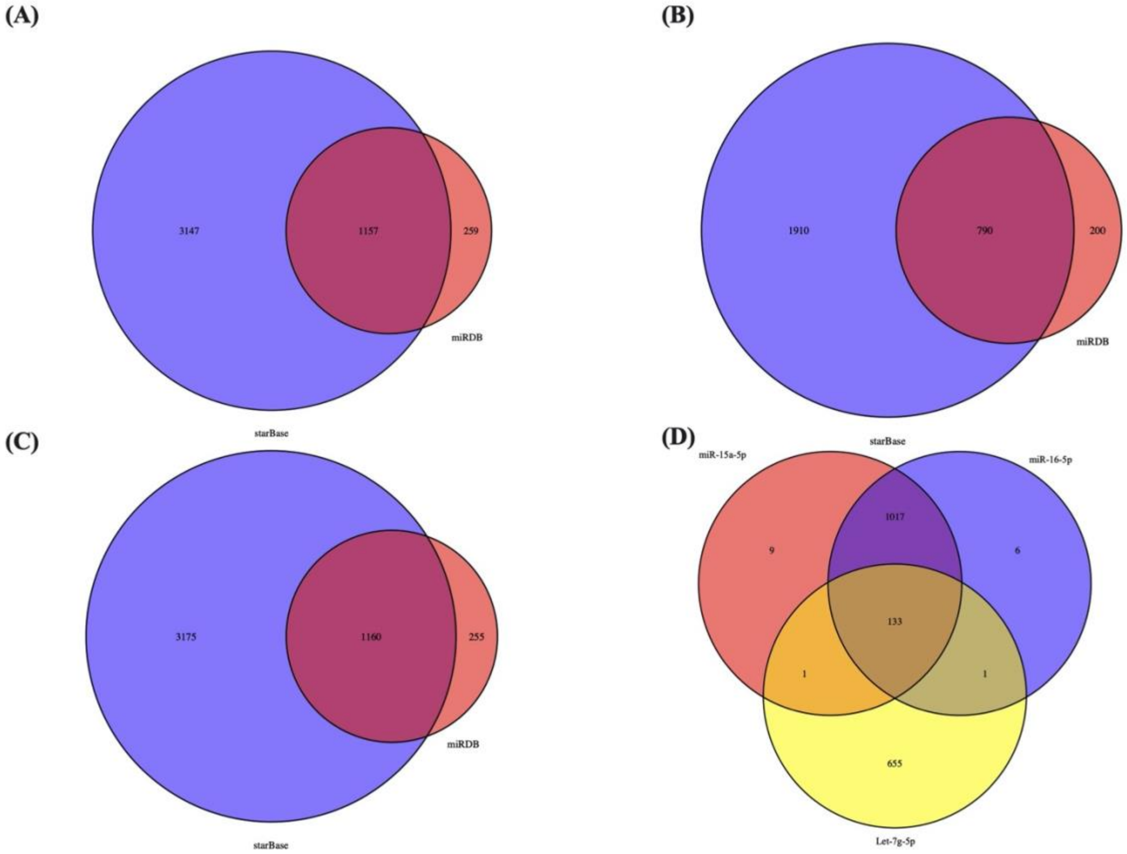

Supplementary Figure 2. Target genes of miR-16-5p, Let-7g-5p, miR-15a-5p were obtained from databases miRDB and starBase. **(A)**A venn diagram of miR-16-5p target genes. **(B)**A venn diagram of Let-7g-5p target genes. **(C)**A venn diagram of miR-15a-5p target genes. **(D)**A venn diagram of three miRNAs target genes.

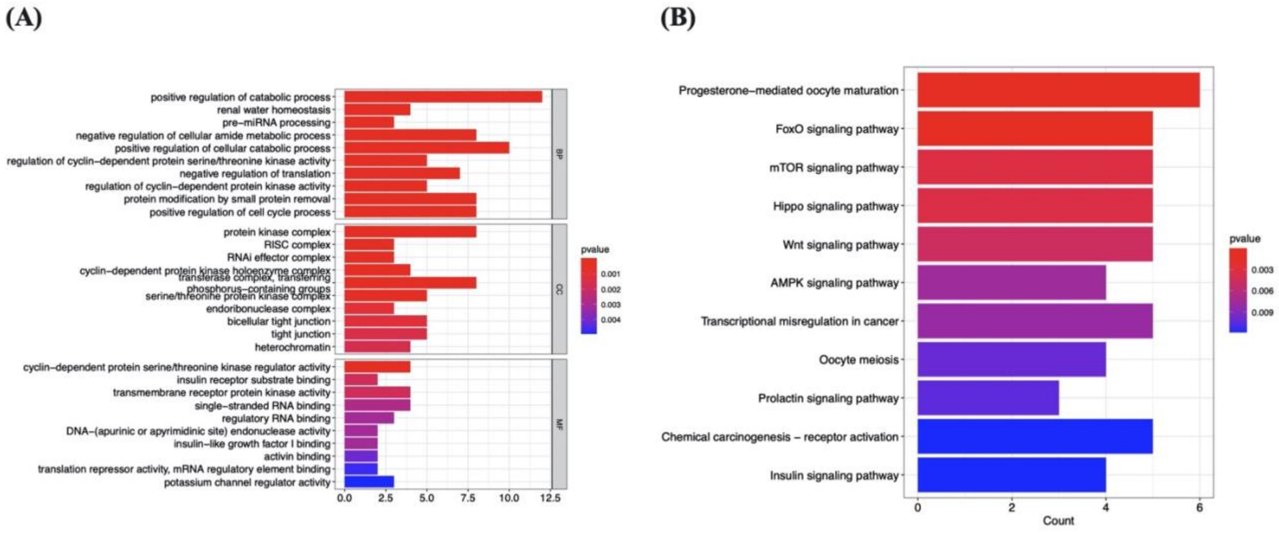

Supplementary Figure 3. GO and KEGG pathway enrichment analysis for predicted miRNA targets. **(A)**Gene ontology in terms of Biological processes (BP), cellular Components (CC), molecular function (MF). **(B)**KEGG pathway analysis.

(A) **(B)**

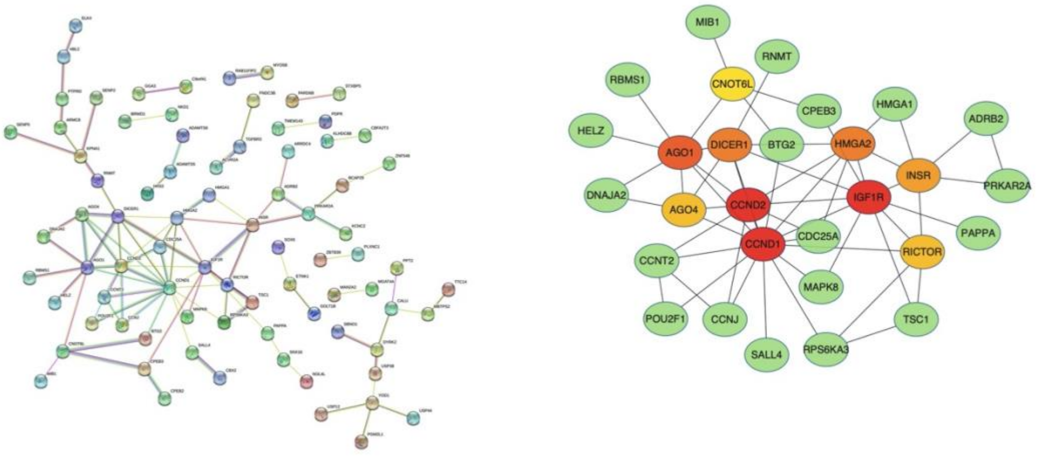

Supplementary Figure 4. PPI network construction and screened hub genes. **(A)** PPI network construction of miRNAs target genes (minimum required interaction score 0.15). **(B)** Visualization of hub genes using the cytoscape plugin, CCND1 is the hub gene.

	**eGFR(ml/min/1.73m^2)**	**Proteinuria(g/day)**	**M**	**E**	**S**	**T**	**C**
**miR-16-5p**	r = -0.144	r = 0.018	r = -0.009	r = 0.164	r = -0.057	r = -0.106	r = 0.129
	p = 0.058	p = 0.811	p = 0.905	p = 0.031*	p = 0.457	p = 0.163	p = 0.089
**Let-7g-5p**	r = -0.150	r = 0.154	r = -0.001	r = 0.086	r = 0.022	r = -0.1113	r = 0.043
	p = 0.048*	p = 0.043*	p = 0.994	p = 0.258	p = 0.776	p = 0.138	p = 0.575
**miR-15a-5p**	r = -0.128	r = -0.001	r = -0.058	r = 0.061	r = -0.015	r = -0.101	r = 0.130
	p = 0.097	p = 0.988	p = 0.451	p = 0.433	p = 0.849	p = 0.192	p = 0.092

Supplementary Table 1. Correlation between the miRNAs and the clinical and pathological indicators.
*, Statistical significance with P< 0.05.
